# Inferring Quantitative Trait Pathways Associated with Bull Fertility from a Genome-Wide Association Study

**DOI:** 10.3389/fgene.2012.00307

**Published:** 2013-01-11

**Authors:** Francisco Peñagaricano, Kent A. Weigel, Guilherme J. M. Rosa, Hasan Khatib

**Affiliations:** ^1^Department of Animal Sciences, University of WisconsinMadison, WI, USA; ^2^Department of Dairy Science, University of WisconsinMadison, WI, USA; ^3^Department of Biostatistics and Medical Informatics, University of WisconsinMadison, WI, USA

**Keywords:** gene ontology, gene-set analysis, genome-wide study, male fertility, quantitative trait pathway

## Abstract

Whole-genome association studies typically focus on genetic markers with the strongest evidence of association. However, single markers often explain only a small component of the genetic variance and hence offer a limited understanding of the trait under study. As such, the objective of this study was to perform a pathway-based association analysis in Holstein dairy cattle in order to identify relevant pathways involved in bull fertility. The results of a single-marker association analysis, using 1,755 bulls with sire conception rate data and genotypes for 38,650 single nucleotide polymorphisms (SNPs), were used in this study. A total of 16,819 annotated genes, including 2,767 significantly associated with bull fertility, were used to interrogate a total of 662 Gene Ontology (GO) terms and 248 InterPro (IP) entries using a test of proportions based on the cumulative hypergeometric distribution. After multiple-testing correction, 20 GO categories and one IP entry showed significant overrepresentation of genes statistically associated with bull fertility. Several of these functional categories such as small GTPases mediated signal transduction, neurogenesis, calcium ion binding, and cytoskeleton are known to be involved in biological processes closely related to male fertility. These results could provide insight into the genetic architecture of this complex trait in dairy cattle. In addition, this study shows that quantitative trait pathways inferred from single-marker analyses could enhance our interpretations of the results of genome-wide association studies.

## Introduction

The decline in reproductive performance in dairy cattle is a serious concern of farmers and the dairy industry worldwide (Lucy, 2001; Pryce et al., 2004). Most reproductive studies in cattle have focused on fertility of the cow, while male fertility has received much less consideration. Nonetheless, some studies have reported that a significant percentage of reproductive failure in dairy cattle is attributable to male subfertility (DeJarnette et al., 2004). Consequently, bull fertility should not be ignored in breeding schemes aimed at improving the reproductive performance of dairy cattle (Braundmeier and Miller, 2001).

A growing body of evidence suggests that male fertility is influenced by genetic factors. For instance, Khatib et al. (2010) reported that polymorphisms in the *FGF2* and *STAT5A* genes are associated with estimated relative conception rate in Holstein bulls. In addition, Li et al. (2012) showed that *MAP1B* and *PPP1R11*, highly conserved spermatogenesis genes, are significantly associated with sire conception rate (SCR) in the Holstein breed. Recently, some studies have addressed bull fertility using a genome-wide association approach. In this sense, Blaschek et al. (2011) identified several genomic regions associated with non-compensatory fertility in Holstein bulls. Fortes et al. (2012a) reported that genomic regions in BTA2, BTA14, and chromosome X are associated with testicular development, sperm quality, and different hormone levels measured throughout puberty in Brahman bulls. Furthermore, Peñagaricano et al. (2012) have identified a panel of single nucleotide polymorphisms (SNPs) associated with SCR in a population of 1,755 Holstein sires. Some of the significant SNPs were located within or near genes affecting male fertility, such as the sperm acrosome reaction, chromatin remodeling during spermatogenesis, or the meiotic process during male germ cell maturation (Peñagaricano et al., 2012). Altogether, these studies have identified genomic regions and individual genes associated with bull fertility.

However, although whole-genome association studies have been successful in detecting genetic variants associated with complex traits such as bull fertility, they identify only the most significant genetic markers because of the stringent statistical criteria necessary for minimizing false positive results. Therefore, the identified SNPs represent only a small fraction of the variants contributing to the complex trait and hence the vast majority of genetic variation remains hidden. Thus, testing only for associations with single SNPs is insufficient to dissect the complex genetic structure of a quantitative trait (Peng et al., 2010).

It is increasingly accepted that pathway-based analysis, an alternative strategy based on testing the association of modules of functionally related genes, can greatly complement single-marker association analyses in understanding genetic determinants and biological pathways affecting complex traits (Wang et al., 2007; Chasman, 2008). In a pathway-based analysis, multiple contributing factors are considered simultaneously, rather than focusing on single markers with the strongest evidence of association with a trait. Therefore, this approach provides a unique opportunity to detect causal pathways and genetic mechanisms underlying complex phenotypes (Weng et al., 2011).

The objective of the present study was to identify biologically relevant pathways affecting SCR as a measure of bull fertility using results of a single-marker association analysis. The identification of fertility pathways would provide better understanding of the genetic architecture of this complex trait in cattle.

## Materials and Methods

In a previous study, we carried out a genome-wide association analysis for SCR in Holstein dairy cattle (Peñagaricano et al., 2012). Here, we used the results of that single-marker association analysis to perform a pathway-based association analysis. The phenotypic and genotypic data and the statistical models used for the single-marker association analysis were described in detail by Peñagaricano et al. (2012) and are reviewed briefly here.

### Phenotypic and genotypic data

A total of 1,755 Holstein bulls with SCR data, a phenotypic evaluation of bull fertility provided by the USDA-ARS Animal Improvement Programs Laboratory (Beltsville, MD, USA), were used in the study. In brief, SCR is the expected difference in conception rate of a sire compared with the mean of all other evaluated sires. SCR values from the 1,755 bulls ranged from −10.66 to +6.80%, and the number of breedings per bull ranged from 303 to 111,402. SCR data were obtained from seven consecutive evaluations between August 2008 and December 2010. For bulls with multiple evaluations, the most recent SCR evaluation was used in the analysis. Genotypes of the 1,755 bulls for the Illumina BovineSNP50 Bead Chip were also provided by USDA-ARS Animal Improvement Programs Laboratory. SNPs with minor allele frequencies below 5% were removed. After data editing, 38,650 markers were available.

### Single-marker association analysis

Associations between SNP genotype and SCR were analyzed by using the SNP allele count as a linear covariate, as well as by using the SNP genotype as a categorical variable. The following mixed model was used to analyze SNP genotype as a linear covariate:

$$\text{SC}{\text{R}}_{ijkl}=\mu +\text{EVA}{\text{L}}_{j}+\beta_{k}\text{SN}{\text{P}}_{ik}+\text{sir}{\text{e}}_{l}+{\text{e}}_{ijkl}$$

where μ is the general mean, EVAL*_j_* is the fixed effect of the *j*^th^ AIPL-USDA SCR evaluation (*j* = 1, 2, …, 7), SNP*_ik_* is the number of copies of one allele of the SNP (corresponding to 0, 1, or 2 copies) carried by the *i*^th^ bull (*i* = 1, 2,…, 1755), β*_k_* is the regression coefficient for the SNP considered (also known as the allele substitution effect), sire*_l_* represents the random additive genetic effect of the *l*^th^ sire (*l* = 1, 2, …, 222) of the *i*^th^ bull, and e*_ijkl_* represents the random residual for each observation. For the analysis using SNP genotype as a categorical variable, the same statistical model was used replacing β*_k_* SNP*_ik_* with SNP*_ik_* where SNP*_ik_* is the fixed effect of the *k*^th^ genotypic class (*k* = 1, 2, 3) for the SNP considered carried by the *i*^th^ bull.

The distributions of sire*_l_* and e*_ijkl_* were assumed as $N(0,A\sigma_{s}^{2})$ and $N(0,W^{-1}\sigma_{e}^{2})$, respectively, where *A* represents the matrix of additive relationships (1,510 × 1,510) between sires in the pedigree and *W* is a diagonal matrix of order 1,755 with its elements representing reliabilities of the SCR values. Associations between SNPs and SCR were tested using a likelihood ratio test, by comparing the aforementioned models to a reduced model without the SNP effect. The analyses were performed using the pedigreemm package (Vazquez et al., 2010) of the R language/environment (R Development Core Team, 2009).

### Assignment of SNPs to genes

The UMD 3.1 bovine genome sequence assembly (Zimin et al., 2009) was used for SNP assignment. SNPs were assigned to genes if they were located within the genomic sequence of an annotated gene (i.e., genomic sequence between the start of the first exon and the end of the last exon) or within 20 kb of the 5′ or 3′ ends of the first and last exon, respectively. The distance of 20 kb was used in order to capture proximal regulatory and other functional regions that may lie outside, but close to, the gene. Additionally, it has been reported that strong linkage disequilibrium can extend up to 20–30 kb in Holstein cattle (de Roos et al., 2008; Kim and Kirkpatrick, 2009). If a SNP was found to be located within or proximal to more than one gene, all of these genes were included in the subsequent analyses. Finally, a gene was considered as significantly associated with SCR if that gene contained at least one SNP with a nominal *P*-value ≤ 0.05.

### Assignment of genes to pathways

Gene Ontology (GO) and InterPro (IP) databases were used to define functional sets of genes. The GO database (Ashburner et al., 2000), which is probably the most popular among the initiatives for defining functional classes of genes, assigns biological descriptors (named GO terms) to genes on the basis of the properties of their encoded products. These terms fall into three types: biological process, molecular function, and cellular component. Genes assigned to the same GO term can thus be regarded as members of a category of genes that are more closely related in terms of some aspect of their biology than are random sets of genes. The IP database is an integrated documentation resource of protein families, domains, and functional sites (Mulder et al., 2003). Applications of the IP database span a range of biologically important areas that include automatic annotation of protein sequences and genome analysis.

### Pathway-based association analysis

The association of a given pathway with SCR was analyzed using a test of proportions based on the cumulative hypergeometric distribution (Tavazoie et al., 1999). This test, also known as Fisher’s exact test and commonly used in the analysis of contingency tables, was performed to search for an overrepresentation of significantly associated genes among all the genes in the pathway. The *P*-value of observing *k* significant genes in the pathway was calculated by

$$P\text{ - value}=1-\overset{k-1}{\underset{i=0}{\sum }}\frac{\left(\;\begin{array}{c} S\\ i \end{array}\right)\left(\;\begin{array}{c} N-S\\ m-i \end{array}\right)}{\left(\;\begin{array}{c} N\\ m \end{array}\right)}$$

where *S* is the total number of genes that are significantly associated with SCR, *N* is the total number of genes that are analyzed in the study, and *m* is the number of genes in the pathway.

To avoid testing overly narrow or broad functional categories, we tested only GO terms with more than 30 genes that were located between levels 5 and 9 in the GO hierarchy. Similarly, we tested only entries in the IP database with more than 30 genes. Furthermore, to account for multiple hypothesis testing (i.e., one for each functional term) and control the false discovery rate, the procedure proposed by Benjamini and Hochberg (1995) was adopted, and the *q*-value for each test was obtained. The *q*-value of a test measures the proportion of false positives incurred when that particular test is deemed significant. In this study, functional categories with a *q*-value ≤ 0.05 were considered significant. All analyses were performed using the procedure FatiGO (Al-Shahrour et al., 2004), implemented on the platform Babelomics (Medina et al., 2010).

## Results and Discussion

In a previous study, we performed a genome-wide association study for bull fertility using a total of 38,650 SNPs spanning the entire bovine genome (Peñagaricano et al., 2012). Associations between SNPs and SCR were analyzed using a mixed linear model that included a random polygenic effect and each SNP genotype, one at a time, as a linear covariate or a categorical variable. The first analysis had more power to detect significant SNPs with additive effects, whereas the second approach allowed the detection of significant markers that show some degree of dominance. Out of the 38,650 genetic markers, 3,353 and 3,096 showed significant associations with SCR (nominal *P*-value ≤ 0.05) using SNP genotypes as linear covariates or categorical variables, respectively. In total, 4,450 different SNPs showed significant associations with bull fertility in one or both models used for the analysis.

The first step in this pathway-based analysis of a whole-genome association study was to assign SNPs to genes. A total of 21,325 of the 38,650 examined SNPs were located within annotated genes or within 20 kb upstream or downstream from annotated genes. This set of SNPs defined a total of 16,819 genes annotated in the UMD 3.1 bovine genome sequence assembly, which in turn were evaluated for the pathway analysis. In addition, 2,767 of the 16,819 genes had one or more SNPs with nominal *P*-value ≤ 0.05; hence, these genes were defined as significantly associated with bull fertility.

The next step in the analysis was to assign the 16,819 genes into pathways. We tested GO categories and IP entries with more than 30 genes and, in the case of GO terms, located between levels 5 and 9 in the GO hierarchy. A total of 662 GO terms and 248 IP entries met these requirements and hence were tested by a hypergeometric test for enrichment of significant genes associated with bull fertility.

Twenty GO terms showed significant overrepresentation of genes statistically associated with SCR. Table 1 shows the 11 GO terms that belong to the biological process domain, and Table 2 displays the nine GO terms classified into the molecular function (4) and cellular component (5) domains, respectively. These tables show the number of genes in each functional category (out of 16,819 genes), the expected number of significant genes under the null hypothesis (i.e., with no significant overrepresentation), and the actual number of significant genes per category. Only GO terms with *q*-value ≤ 0.05 were considered significant. In addition, one entry of the IP database, *Haloacid dehalogenase-like hydrolase* (IPR005834) showed significant enrichment (*q*-value = 0.027) of genes associated with bull fertility. This entry, with a total of 37 genes, showed 16 significant genes, which is 10 more than expected under the null hypothesis. Genes statistically associated with SCR (nominal *P*-value ≤ 0.05) within each significant category (i.e., the 20 GO terms and IPR005834) are listed in File S1 in Supplementary Material.

**Table 1:** **Biological process terms significantly overrepresented with genes statistically associated with bull fertility**.

GO ID	Term (GO hierarchy level)	No. genes in the GO term	Expected no. significant genes	Actual no. significant genes	*q*-Value
0007242	Intracellular signaling cascade (6)	889	146	187	0.032
0007264	Small GTPase mediated signal transduction (7)	352	58	83	0.035
0007265	Ras protein signal transduction (8)	171	28	48	0.032
0007266	Rho protein signal transduction (9)	91	15	27	0.048
0046578	Regulation of Ras protein signal transduction (9)	145	24	39	0.046
0051056	Regulation of small GTPase mediated signal transduction (8)	182	30	47	0.046
0022008	Neurogenesis (6)	325	53	75	0.048
0030182	Neuron differentiation (8)	269	44	65	0.046
0048666	Neuron development (9)	201	33	53	0.034
0032940	Secretion by cell (6)	171	28	45	0.046
0046903	Secretion (5)	197	32	50	0.046

**Table 2:** **Molecular function and cellular component terms significantly overrepresented with genes statistically associated with bull fertility**.

GO ID	Term (GO hierarchy level)	No. genes in the GO term	Expected no. significant genes	Actual no. significant genes	*q*-Value
**MOLECULAR FUNCTION**					
0005509	Calcium ion binding (5)	525	86	118	0.018
0016462	Pyrophosphatase activity (6)	578	95	126	0.020
0016818	Hydrolase activity, acting on acid anhydrides (5)	581	96	127	0.018
0017111	Nucleoside-triphosphatase activity (7)	563	93	122	0.022
**CELLULAR COMPONENT**					
0005856	Cytoskeleton (8)	599	99	124	0.048
0015629	Actin cytoskeleton (9)	185	30	46	0.045
0044420	Extracellular matrix part (5)	65	11	21	0.040
0044430	Cytoskeletal part (9)	373	61	85	0.039
0044459	Plasma membrane part (6)	678	112	138	0.047

Six of the 11 significant GO terms classified into the biological process domain show a very close relationship in the GO hierarchy (Table 1). Indeed, *Rho protein signal transduction* (GO:0007266) is part of *Ras protein signal transduction* (GO:0007265) and *Regulation of Ras protein signal transduction* (GO:0046578) is part of *Regulation of small GTPase mediated signal transduction* (GO:0051056). Furthermore, *Ras protein signal transduction* is part of *Small GTPase mediated signal transduction* (GO:0007264), which in turn is part of *Intracellular signaling cascade* (GO:0007242). Interestingly, GO:0046578 and GO:0051056 negatively regulate GO:0007265 and GO:0007264, respectively. Overall, these results point out that an intracellular signaling pathway involving small GTPases and their negative regulators was significantly associated with bull fertility. Interestingly, a recent microarray study showed that *Regulation of small GTPase mediated signal transduction* term was significantly enriched with genes differentially expressed between human sperm that achieved successful fertilization and sperm that failed the intracytoplasmic sperm injection (García-Herrero et al., 2011).

Small GTPases coordinate diverse cellular functions, including actin reorganization, junction dynamics, cell movement, cell cycle, cell transformation, and gene transcription (Bustelo et al., 2007). Several recent studies have shown that small GTPases are relevant molecules for spermatogenesis. For instance, some studies have reported that the translocation of elongating/elongate spermatids across the seminiferous epithelium is assisted by small GTPases present at the site of the ectoplasmic specialization, a unique anchoring junction type located in the testes (Lui et al., 2003). In addition, other studies have shown that small GTPases are involved in regulation of the mammalian acrosome reaction by controlling the membrane fusion system in the sperm (Iida et al., 1999). The acrosome reaction allows spermatozoa to penetrate the zona pellucida and fuse with the oocyte membrane and, hence, this is a crucial step during the fertilization (Brucker and Lipford, 1995). Overall, these results provide further evidence for the possible role of small GTPases and their regulators in spermatogenesis and, hence, in male fertility.

Three significant GO terms classified into the biological process domain are related to neurongenesis (Table 1). In fact, *Neuron development* (GO:0048666) is part of *Neuron differentiation* (GO:0030182), which in turn is part of *Neurogenesis* (GO:0022008). One well-known connection between neurons and male fertility is provided by the hypothalamic–pituitary–gonadal axis. Indeed, the maintenance of spermatogenesis depends upon stimulation of the testes by the gonadotropic hormones, follicle-stimulating hormone, and luteinizing hormone, both produced by the pituitary gland in response to gonadotropin-releasing hormone from the hypothalamus (Zirkin, 1998). It is important to note that recent studies have reported *de novo* neurogenesis in postnatal and adult mammalian hypothalamus (Pierce and Xu, 2010). Another well documented connection between neurogenesis and spermatogenesis is the fact that these two biological processes share several key molecular regulators. Heat shock factors and proteins, bone morphogenetic proteins, and inhibitor of DNA-binding/differentiation (Id) proteins, among others, play relevant roles in both processes (Yokota, 2001; Abane and Mezger, 2010; Björk and Sistonen, 2010; Bragdon et al., 2011). Heat shock factors, a family of transcriptional regulators, trigger the expression of genes encoding proteins (i.e., heat shock proteins) that function as molecular chaperones, contributing to establish a cytoprotective state in response to different stress conditions (Björk and Sistonen, 2010). In addition to promoting cell survival under stressful conditions, heat shock factors and proteins are important for developmental processes such as spermatogenesis and neurogenesis (Abane and Mezger, 2010). Bone morphogenetic proteins, potent growth factors belonging to the transforming growth factor beta superfamily, are responsible for many biological processes including embryogenesis, neurogenesis, and spermatogenesis (Bragdon et al., 2011). Id proteins comprise a family of proteins that are positive and negative regulators of proliferation and differentiation, respectively (Yokota, 2001). Id proteins are key regulators of several developmental processes such as neurogenesis, lymphoid organogenesis, mammary gland development, and spermatogenesis (Yokota, 2001). Altogether, these results present further evidence of the tight connection between neurogenesis and male fertility.

Four GO terms classified into the molecular function domain showed significant association with bull fertility (Table 2). Three of these GO terms showed a very close relationship in the GO hierarchy: *Nucleoside-triphosphatase activity* (GO:0017111) is part of *Pyrophosphatase activity* (GO:0016462), which in turn is part of *Hydrolase activity, acting on acid anhydrides, in phosphorus-containing anhydrides* (GO:0016818). Nucleoside-triphosphatases are enzymes that catalyze the hydrolysis of a nucleoside triphosphate in a nucleoside diphosphate and phosphate. Pyrophosphatases are enzymes that catalyze the hydrolysis of a pyrophosphate bond between two phosphate groups. Both types of enzymes, which belong to the family of hydrolases, participate in purine and thiamine metabolism (Zöllner, 1982). Interestingly, Sertoli cells, which are located in the seminiferous tubules and play an essential role in the maintenance and control of spermatogenesis, have shown remarkable activity of these two enzymes (Casali et al., 2001). In fact, these enzymes play an important role in the physiological control of extracellular levels of nucleotides and nucleosides inside the seminiferous tubules, which in turn can modulate Sertoli cell responses (Casali et al., 2001). Thus, findings of this study provide further support for the relevant role of these enzymes (i.e., nucleoside-triphosphatases and pyrophosphatases) in spermatogenesis.

The *calcium ion binding* (GO:0005509) term showed a significant overrepresentation of genes associated with bull fertility (Table 2). The relationship between calcium and sperm physiology is well documented. Mammalian spermatozoa possess multiple voltage-gated calcium channels and use calcium ion signals to control several physiological responses (Darszon et al., 1999). Indeed, calcium is considered a regulator of sperm motility, a participant in capacitation, and an essential second messenger for the acrosome reaction (Wennemuth et al., 2003). Therefore, our findings provide more evidence of the strong association between calcium and sperm biology and, hence, male fertility.

Three of the five significant GO terms grouped within the cellular component domain are related to the cytoskeleton biology. Indeed, *Actin cytoskeleton* (GO:0015629) and *Cytoskeletal part* are part of *Cytoskeleton* (GO:0005856). The close relationship between cytoskeleton and spermatogenesis is well documented. Sertoli cells are characterized by their well-developed cytoskeleton, which is responsible for the collective organization of the seminiferous epithelium (Mruk and Cheng, 2004). Morphological studies have shown that the Sertoli cell cytoskeleton, among other functions, positions, anchors, and aids in the movement of developing germ cells and participates in the release of mature spermatids from the seminiferous epithelium at spermiation (Mruk and Cheng, 2004). In fact, the different cellular events that occur during spermatogenesis are associated with extensive changes in cell shape and size and germ cell movement, in which the cytoskeleton has a key role (Lie et al., 2010). Interestingly, García-Herrero et al. (2010) have reported that the *Cytoskeleton* term was significantly enriched with genes differentially expressed between sperm of infertile and fertile human males. Therefore, our study provides further evidence of the close relationship between cytoskeletal dynamics and spermatogenesis.

The terms *Secretion by cell* (GO:0032940), *Secretion* (GO:0046903), (Table 1) and *Extracellular matrix part* (GO:0044420), and *Plasma membrane part* (GO:0044459; Table 2) also showed significant enrichment of genes statistically associated with bull fertility. Despite the wide range of functions of these categories, it is worth noting that spermatogenesis and fertilization involve biological processes related to these terms. For instance, many of the functions of the Sertoli cells are carried out by their protein secretions: these cells synthesize and secrete a number of proteins that have functions such as hormone-like or growth factor-like activity, transport functions, enzymatic activities, as well as proteins that contribute to the basement membrane (Griswold, 1998). In addition, it seems that the plasma membrane plays an important role in Sertoli-Sertoli and Sertoli-germ cell interactions (Mruk and Cheng, 2004). Also, it has been reported that the extracellular matrix regulates Sertoli cell differentiation and germ cell development (Hadley et al., 1985).

InterPro is a database that integrates diverse information about families, domains, and functional sites of proteins. One entry of the IP database, *Haloacid dehalogenase-like hydrolase* (IPR:005834), showed significant overrepresentation of genes associated with SCR. IPR:005834 entry contains a group of hydrolase enzymes including l-2-haloacid dehalogenase, epoxide hydrolases, and phosphatases. These enzymes are involved in diverse cellular functions such as membrane transport, signal transduction, metabolism, and DNA repair (Arand et al., 2003). Interestingly, Iguchi et al. (2006), studying gene expression profile in meiotic male germ cells, reported that one haloacid dehalogenase-like hydrolase is highly expressed in the testes. Thus, our finding provides further evidence of the possible involvement of these enzymes in male fertility.

Previous studies have derived gene networks and pathways related to complex traits in livestock species. For instance, Fortes et al. (2012b) have used genome and trait associations, hypothalamic-transcriptome data and transcription factors in order to find candidate genes and gene–gene interactions related to first service conception in Brangus heifers. Moreover, Lewandowska-Sabat et al. (2012) have integrated genomic and gene expression data aimed at identifying significant pathways involved in the immune response to mastitis in dairy cattle. Here, we have extended our previous genome-wide association study to identify biologically relevant pathways involved in bull fertility. Previously, we have reported eight SNPs associated with SCR with a genome-wise adjusted *P*-value below 0.10 (Peñagaricano et al., 2012). Thus, beyond the enumeration of possible candidate genes (i.e., genes harboring the most significant genetic markers) done in our previous report, in this study we have performed a gene-set enrichment analysis to characterize quantitative trait pathways related to bull fertility. The identified pathways could contribute to a better understanding of the genetic architecture of male fertility in dairy cattle. Moreover, as discussed by Snelling et al. (2012), genetic markers within and surrounding genes initially identified by gene-set enrichment analysis may facilitate the incorporation of marker-assisted selection for complex traits in commercial breeding schemes.

One potential limitation of this study is the limited number of GO categories and IP entries that were analyzed. To avoid testing overly narrow or broad functional categories, while at the same time address the multiple-testing issue, we have analyzed only functional categories with more than 30 genes and, in the case of GO terms, those that were located between levels 5 and 9 in the GO hierarchy. As a result, a total of 662 GO categories and 254 IP entries were interrogated. It is important to note that a key factor in these analyses is the total number of genes, which determines the total number of functional categories that can be inferred. Here, using data from 38,650 SNPs spanning the entire bovine genome, we could analyze a total of 16,819 annotated genes. Future studies, however, could analyze a larger numbers of genes using a high-density SNP chips (e.g., Illumina Bovine HD 770K), which in turn will increase the number of functional categories and provide even further insight into the genetic architecture of bull fertility.

## Conclusion

In this study, we used genome-wide association SNP results to infer quantitative trait pathways associated with bull fertility. We have identified 20 GO categories and one IP entry that showed significant overrepresentation of genes statistically associated with SCR in Holstein dairy cattle. Interestingly, several of these functional categories, such as small GTPases mediated signal transduction, neurogenesis, calcium ion binding, and cytoskeleton are involved in biological processes closely related to male fertility. These findings could contribute to a better understanding of the genetic architecture of this complex trait in dairy cattle. In addition, pathways enriched with genes that are significantly associated with male fertility could be used in marker-assisted selection in dairy cattle breeding schemes. Thus, the quantitative trait pathway approach used in this study could complement genome-wide association studies for investigating quantitative traits in livestock species.

## Conflict of Interest Statement

The authors declare that the research was conducted in the absence of any commercial or financial relationships that could be construed as a potential conflict of interest.

## Supplementary Material

The Supplementary Material for this article can be found online at http://www.frontiersin.org/Livestock_Genomics/10.3389/fgene.2012.00307/abstract
